# Evaluating Healthcare Center Staff Attitudes Toward Adoption of Tobacco-Free Policies and Concurrent Tobacco Use and Behavioral Health Treatment in Rural and Medically Underserved Areas of Texas

**DOI:** 10.3390/healthcare14203482

**Published:** 2026-10-16

**Authors:** Tarik D. Goulbourne, Isabel Martinez Leal, Maggie Britton, Tzuan A. Chen, Abigail E. Bergey, Ammar D. Siddiqi, Teresa Williams, Kathleen Casey, Lorraine R. Reitzel

**Affiliations:** 1 Department of Psychological, Health, and Learning Sciences, University of Houston, McElhinney Hall, 3623 Cullen Blvd., Houston, TX 77204, USA; tdgoulbo@cougarnet.uh.edu (T.D.G.); tchen3@central.uh.edu (T.A.C.);; 2 Department of Behavioral Science, The University of Texas MD Anderson Cancer Center, 1155 Pressler Street, Houston, TX 77030, USA; brittonm@njhealth.org (M.B.); lreitzel@mdanderson.org (L.R.R.); 3 Department of Health Initiatives, National Jewish Health, 1400 Jackson Street, Denver, CO 80206, USA; 4 HEALTH Research Institute, University of Houston, 4349 Martin Luther King Blvd., Houston, TX 77204, USA; 5 School of Medicine, University of California, 533 Parnassus Ave., San Francisco, CA 94143, USA; 6 Integral Care, 1430 Collier St., Austin, TX 78704, USAkathleen.casey@integralcare.org (K.C.)

**Keywords:** rural populations, social determinants of health, tobacco, cessation, smokefree policies, health disparities, cancer prevention, health, well-being

## Abstract

Background: People with behavioral health conditions (BHCs), including substance use disorders (SUDs) and mental health conditions, use tobacco at elevated rates, yet tobacco management is often overlooked during healthcare visits. Tobacco-free workplace policies (TFWPs) effectively reduce tobacco use in healthcare centers (HCs), but staff valuation is key to successful adoption. Little is known about the factors influencing staff (providers’ and managers’) value of TFWPs, especially in HCs serving rural or medically underserved areas (MUAs). This study examined associations between healthcare providers’ attitudes toward treating tobacco and BHCs simultaneously and their valuation of TFWPs in HCs serving patients in rural/MUAs of Texas. Methods: HCs were enrolled continuously from August 2021 through January 2024, a timespan that primarily fell within the COVID-19 pandemic period (January 2020–May 2023). Generalized linear mixed models assessed links between treatment attitudes and perceived value of TFWPs among 347 providers across 19 HCs serving over 100,000 patients annually, controlling for existing TFWPs in 10 HCs. Rapid qualitative analysis of 16 individual (*n* = 2) and group (*n* = 14) interviews from 12 HCs, including 5 medical healthcare centers and 7 substance use treatment centers, was conducted. Results: Quantitative findings indicated that providers who believed concurrent treatment improved BHCs were more likely to endorse TFWPs as promoting clean and safe environments (OR = 3.81, CI: 2.31–6.28) and as helping staff and patients quit smoking (OR = 3.04, CI: 1.90–4.87). Providers who supported concurrent quitting were also more likely to agree that TFWPs support smoking cessation (OR = 1.77, CI = 1.08–2.92), but this attitude was not associated with endorsing TFWPs as promoting clean and safe environments (OR = 1.25, CI = 0.74–2.11). Qualitative themes: (1) prioritization of “harm reduction” strategies, (2) limited support for tobacco use screening and tobacco cessation services, and (3) hesitation to integrate tobacco-free workplace programs. Integrative findings revealed ambivalent support for concurrent treatment amidst limited resources, provider concerns about concurrent treatment jeopardizing BHC recovery, and mixed reactions to TFWP implementation, particularly concerning patient autonomy, stigma, and practicality. Conclusions: Findings highlight structural and attitudinal barriers to treating tobacco use and BHCs and implementing TFWPs in rural/MUA HCs. Improving providers’ access to tailored education, evidence-based tools, and organizational support can enhance tobacco use treatment and TFWP implementation in rural and MUAs of Texas.

## 1. Introduction

Cigarette smoking among adults in the United States (U.S.) has declined notably over the past decades [1,2], reaching a historical low of 9.9% among adults in 2024 [3]. Despite this progress, overall tobacco use continues to exact a heavy toll, remaining the leading preventable cause of death and disability in the U.S. and disproportionately affecting certain populations [4]. People with behavioral health conditions (BHCs; i.e., substance use disorders and/or mental health disorders) continue to use tobacco at high rates, contributing to elevated morbidity and premature mortality [4]. As many as 65–85% of people in treatment for substance use disorders [5,6] smoke cigarettes—the most common form of tobacco use—and people with histories of mental illness smoke at rates at least 1.8 times higher than people without histories of mental illness [4,7,8,9,10,11]. Although interest in quitting tobacco among people receiving treatment for BHCs is comparable to that of the general population [7,12,13,14,15,16,17], behavioral health and medical treatment centers do not consistently address patients’ tobacco use [18].

The Centers for Disease Control and Prevention’s (CDC) recommendations for best practices in comprehensive tobacco control emphasize that tobacco-free policies are essential for reducing tobacco use and exposure to secondhand smoke [19]. Further, the United States Preventive Services Task Force and Clinical Practice Guidelines recommend screening for tobacco use at every patient encounter and providing behavioral interventions, such as counseling and pharmacotherapy, to patients who use tobacco [20,21]. Despite recommendations that all tobacco users—including those with BHCs—receive the same evidence-based interventions, and despite evidence that people with BHCs who use tobacco want to quit, can quit, and that quitting benefits their BHC outcomes, implementation of tobacco treatment remains low in these settings [21,22]. National data from 2023 showed that while most healthcare centers (HCs; i.e., medical healthcare centers and substance use treatment centers) screened for tobacco use, only 53.1% of mental health facilities and 69.9% of substance use facilities offered cessation counseling services. Less than half of all HCs provided nicotine replacement therapy (NRT), with mental health facilities making up 35% and substance use facilities 40.2%, or non-nicotine cessation medications, with mental health facilities comprising 33.6% and substance use facilities 35.3% [18]. Moreover, only 53.9% of mental health facilities and 33.9% of substance use facilities had tobacco-free policies [18]. Additionally, healthcare centers, particularly in rural areas where reduced access to tobacco cessation resources is a significant barrier to treatment, show a similar pattern. Prior work indicates that, among HCs in rural parts of Texas, while providers in medical healthcare centers (i.e., Federally Qualified Healthcare Center Look-Alike facilities providing primary care and prevention services to underserved populations) reported higher rates of asking and advising clients about tobacco cessation than providers in substance use treatment centers, providers across both settings reported feeling unprepared to adequately help patients quit tobacco [23]. These findings indicate improvements in the provision of tobacco cessation treatment practices and policies since the prior 2016 report [24], but highlight areas for further development, particularly regarding pharmacotherapy (half of the evidence-based guidelines), provider training for facilitating tobacco cessation treatment, and tobacco-free policy implementation.

Tobacco cessation is associated with a 25% increase in sobriety among people receiving treatment for substance use disorder [25,26], and smoking cessation, in particular, is associated with decreases in reported anxiety, depression, and stress [25,27]. Still, within substance use treatment settings (i.e., Federally Qualified Health Centers, Local Mental Health Authorities (LMHAs), substance use treatment programs in LMHAs, stand-alone substance use treatment centers, community-based opiate treatment programs, inpatient drug and alcohol services, detoxification units, and outpatient hospital-based drug and alcohol services), the provision of tobacco cessation treatment has historically been limited due to myriad barriers, including a lack of provider training and confidence in treating tobacco dependence, as well as a lack of time and resources to provide patient cessation care [28,29]. Furthermore, long-standing misconceptions—such as beliefs that people with BHCs are not interested in quitting or are unlikely to succeed, and that addressing tobacco use could interfere with substance use treatment outcomes and long-term recovery—continue to shape provider attitudes and contribute to persistent treatment barriers [28,29]. Similarly, within mental health settings, various factors have contributed to shaping the culture of normalizing tobacco use and presented challenges to the provision of tobacco cessation treatment. These factors include mental health professionals’ beliefs about their clients’ abilities and willingness to quit, their prioritization of mental health treatment [30], and efforts by the tobacco industry and mental health patient advocates to counter smoking bans in inpatient settings [31].

Yet, people with BHCs are more likely to die from their tobacco use than their substance use disorders [32,33,34]. Indeed, tobacco use has a higher mortality rate than alcohol and all other substances, murders and suicides, and car accidents, combined, with cigarette smoking causing more than 490,000 deaths annually in the U.S. [4], impacting the health of over 16 million people living with tobacco-related illnesses [35]. Further elucidating this point, tobacco-related concerns account for approximately 47% of deaths among people with serious mental health conditions [36] and 53% of deaths among people with substance use disorders (SUDs) [37]. The disparities in tobacco use and tobacco-related deaths among people with BHCs emphasize an ongoing need for targeted tobacco control programs and interventions [32,38,39].

Tobacco-free workplace programs that integrate tobacco use interventions, including screening and cessation treatment, with the implementation of tobacco-free workplace policies (TFWPs), offer an evidence-based path toward addressing treatment gaps in HCs. The organizational readiness for change (ORC) theory provides a framework for understanding and addressing systemic and cultural barriers to implementing tobacco-free workplace programs, inclusive of TFWPs. ORC posits two factors that influence an organization’s capacity to make changes: change valence—the value members place on a particular change—and change efficacy—the members’ shared beliefs about their capabilities to implement the change [40]. Change valence is largely impacted by organizational members’ values and commitment to change [40]. While an organization’s members do not need to have the same reasons for valuing the proposed change, a collective valuing of the change is necessary for successful implementation. Using the ORC as a guiding theory, understanding healthcare providers’ attitudes toward TFWPs and tobacco cessation care can help identify ways to enhance motivation and commitment to implementing necessary change.

Few studies have examined associations among provider attitudes, TFWPs, and the implementation of tobacco cessation care in HCs. Even fewer have examined these associations in rural and medically underserved areas (MUAs), where organizational-level barriers are compounded by systemic and regulatory barriers, including insufficient training on tobacco use and cessation treatment approaches, pervasive misunderstandings of tobacco cessation treatment effectiveness among providers, insufficient use of health information technology, and limited time and reimbursement for providers to deliver tobacco cessation treatment [41]. Yet the HCs serving these communities, often one of the few centers offering medical and substance use treatment in their areas, receive little focus in targeted tobacco treatment implementation research and in comprehensive tobacco control efforts, such as tobacco-free workplace programs, inclusive of TFWPs. This lack contributes to higher reported rates of tobacco use and limited access to evidence-based tobacco treatment approaches [42].

Existing research has identified several provider-level barriers to implementation of tobacco-free workplace programs, including providers prioritizing non-nicotine substance use and mental health treatment above tobacco use treatment, providers lacking incentives for delivering tobacco use treatment, and efforts from the tobacco industry to combat TFWPs by providing cigarettes to facilities and funding research supporting the use of tobacco for self-medication among patients with psychiatric illnesses [24]. Providers in rural behavioral health settings express misconceptions about concurrent treatment of tobacco and non-tobacco-related substance use, beliefs that patients were not interested in quitting or reducing tobacco use, and beliefs that tobacco use was not a serious addiction [28]. Many also cited a lack of relevant education and resources to address tobacco use within their centers [28]. A notable theme in this study was the endorsement of the “blame the victim” attitude, which stigmatizes patient health behaviors by placing sole responsibility on patients for tobacco cessation, while overlooking contextual factors (e.g., social determinants of health) impacting the development of health conditions [28]. Additionally, participants noted that patients use tobacco to cope with stress, leading to hesitation around encouraging concurrent treatment due to concerns that it would overwhelm patients [28]. Providers’ reluctance to initiate discussions on tobacco use treatment also acted as a barrier to treatment for patients [28]. Separate studies have found providers’ own tobacco use to be an additional barrier to TFWP implementation and tobacco use treatment, as providers who smoked were more reluctant to discuss nicotine dependence or prioritize tobacco as a treatment goal [43,44]. Moreover, additional research has found that mental health providers also view smoking with patients as a tool to enhance the therapeutic relationships and manage behavior in inpatient settings [45,46]. These findings highlight the complexities in provider attitudes toward tobacco use treatment and how provider attitudes and behaviors can shape tobacco use treatment practices and TFWP implementation.

In HCs serving rural and MUAs, these provider-level barriers are likely exacerbated by structural conditions specific to these settings, such as geographic isolation, workforce shortages, and limited organizational resources. Workforce shortages may mean fewer staff (i.e., direct service providers and managers) available to cross-train on tobacco cessation protocols or to provide tobacco use treatment, while limited organizational resources may reduce the HC’s capacity to stock and distribute evidence-based treatments, such as NRTs. Understanding how these layered barriers affect tobacco control efforts, such as tobacco-free workplace programs, inclusive of TFWPs, is essential to improving implementation in rural communities and MUAs, where people have higher rates of poor health outcomes related to tobacco use and an 18% to 20% higher rate of lung cancer deaths compared to people living in urban areas [47].

Provider attitudes toward tobacco use treatment influence treatment prioritization and the implementation of systemic efforts, such as TFWPs, which support clean and safe environments for care and are associated with reductions in tobacco use [48]. Despite this evidence and treatment guidelines supporting the integration of tobacco cessation treatment in HCs [22], disparities in tobacco cessation treatment persist due to lack of provider education, misconceptions of patients’ ability to quit, personal tobacco use, institutional cultures, and devaluing of tobacco use treatment [12,24,30,31,49]. These provider-level factors reinforce structural barriers to tobacco use treatment provision for patients with BHCs interested in receiving treatment and highlight the need to enhance organizational readiness for change. The present study aims to use a mixed-methods design to (1) examine the relationship between providers’ attitudes toward concurrent treatment of tobacco use and BHCs and their perceived value of TFWPs for creating clean, safe environments and supporting patients and staff in quitting smoking, (2) examine the relationship between providers’ attitudes toward concurrent quitting of tobacco and other substances and their perceived value of TFWPs for creating clean, safe environments and helping patients and staff quit smoking, and (3) identify prevalent themes and subthemes that underpin the providers’ attitudes to contextualize and clarify quantitative results. By examining provider influences, this research aims to identify key factors that may facilitate or hinder the adoption of TFWPs and comprehensive tobacco treatment in HCs, offering insight into how to enhance HC staff value and organizational uptake.

## 2. Materials and Methods

### 2.1. Institutional Approvals

The study was initially approved by the Institutional Review Board (IRB) at the University of Houston (STUDY00002885, initial approval 20 April 2021). However, due to a transition in university affiliation for several members of the project team, the work was paused for three months while the grant was transferred. During this period (October 2022–January 2023), there was no interaction with partners. Subsequent approval was then granted by the Quality Improvement Assessment Board (QIAB; study ID 930, initial approval 21 November 2022) at The University of Texas MD Anderson Cancer Center (MD Anderson) and work with partners resumed. MD Anderson determined that the project did not meet the regulatory definition of human subjects research and thus did not require IRB review, but rather required QIAB approval, as it was focused on quality improvement in healthcare.

### 2.2. Study Design

**Figure 1:**
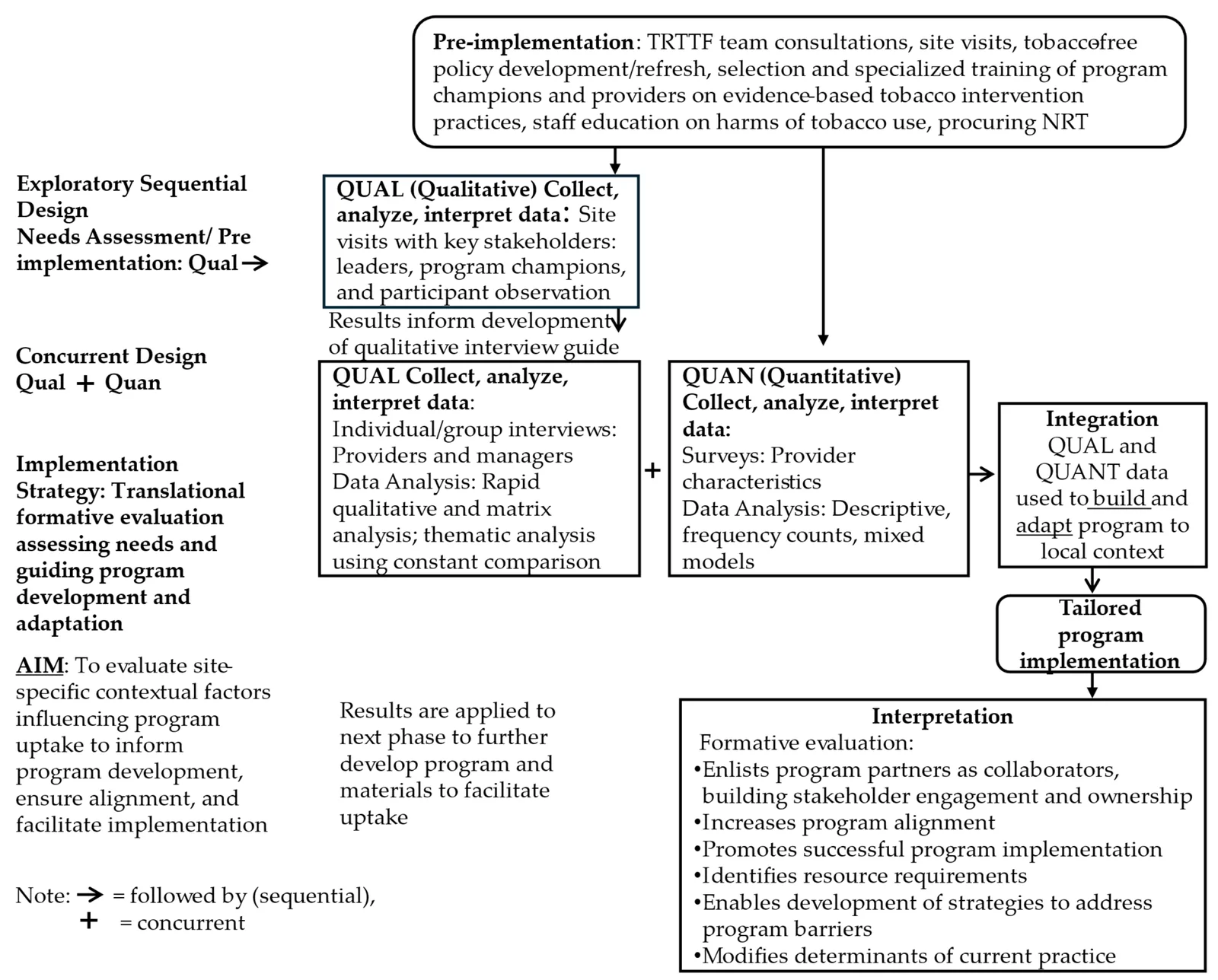
Pre-implementation mixed-methods design to inform implementation of a tobacco-free workplace program in rural Texas. Note: TRTTF = Taking Rural Texas Tobacco-Free; NRT = nicotine replacement therapy. Adapted from: Bergey et al., Provider Perspectives on a Tobacco-Free Workplace Program in Healthcare Settings Serving Rural and Medically Underserved Areas of Texas: A Mixed-Methods Study on Perceived Resource Availability and Value [53].

This study reports findings from Phase 1 of a mixed-methods, multistage evaluation project consisting of 3 phases: pre-implementation, program implementation, and post-implementation [50]. In keeping with Creswell’s (2021) recommendations [51], an exploratory sequential design was used for Phase 1, the pre-implementation, formative evaluation stage [52], the purpose of which was to assess HC partners’ unique characteristics and needs as well as barriers regarding implementation of a comprehensive tobacco-free workplace program in order to tailor the program to centers’ needs in Phase 2, program implementation. As with other advanced mixed-methods designs, the separate phases or studies that comprise the overall evaluative project can be either qualitative, quantitative, or mixed methods [51]. The research team chose to adapt the exploratory sequential design to our research aims for this phase, starting with qualitative data collection (not presented in the current manuscript) and then collecting qualitative and quantitative data concurrently, as each method captures different aspects of the same phenomena; together, they yielded a more comprehensive understanding of local contexts at pre-implementation. Figure 1 depicts how we adapted this study typology according to research aims. Identifying and understanding the contextual factors that can affect implementation are critical to assist in tailoring the program to local contexts, thus enhancing the likelihood of successful uptake in particular settings.

Qualitative data were collected first through participant observation conducted by implementation research team members, our health education specialists, through site visits to participating centers. Summaries of these visits were written up, shared and discussed with the research team, and analyzed by the qualitative researcher to inform writing up of the interview guide by including additional probing questions during the pre-implementation interviews in response to local contexts. The findings from these site visits are beyond the scope of this manuscript but are described here to provide broader context for the study’s exploratory sequential design. Pre-implementation individual and group interviews with staff (including providers and managers) were then conducted, as part of a concurrent component within the larger multiphase evaluation, focusing on contextual factors that impact program implementation, such as attitudes toward implementing tobacco-free workplace programs, inclusive of TFWPs, and concurrent treatment, and any anticipated barriers or facilitators to program adoption. Anonymous surveys of providers and managers were also conducted at roughly the same time that assessed current TFWPs and practices for screening and treating tobacco dependence, as well as beliefs about treating SUDs and tobacco use concurrently. As the research team chose to collect both qualitative and quantitative data for the exploratory phase, these data were independently collected and analyzed by respective analysts who were blinded to results of the other dataset until merging occurred through comparison and synthesizing during the final analysis. Our rationale for blinding of respective analysts was to ensure neutrality or objectivity of findings in each dataset. The rationale for adopting a mixed-methods design for this study was *complementarity* and *development*. In the former, qualitative findings are used to enhance or elaborate upon quantitative results to extend the capabilities of each component and provide a more comprehensive understanding of implementation issues than either method could alone [54], as each component assesses different aspects of the same phenomena [55]. In the latter, both the qualitative data on site-specific contextual data that could hinder or enhance implementation and the quantitative baseline data were merged to inform the development of the intervention.

While the data reported here are from the same dataset (i.e., the same surveys and interviews with the same healthcare centers) and cover the pre-implementation phase of the TRTTF initiative, that reported by Bergey et al. [53], this is a separate study with distinct research aims and variables of interest that measured associations between different variables, resulting in distinct findings. Although both studies shared the same interviews, the aims guiding qualitative data analyses were distinct, and focused on different constructs and segments of the data, thus yielding different results. Though lack of training and provider citing patient use of tobacco as a coping skill while in recovery for other substances featured as themes or subthemes in both studies, their mention served to highlight different implementation challenges within the context of each study. The focus of the Bergey et al. study was on how perceived valuing of TFWPs and treatment of tobacco dependence and resource availability might impact program implementation. In the current study, analysis centered on how these themes related to or influenced provision of concurrent treatment. The only survey variables of interest shared between the two studies were two items on provider valuing of TFWPs (e.g., ‘TFWPs provide clean and safe environments for staff and patients,’ and ‘TFWPs help patients and staff quit smoking’). However, Bergey et al. [53] measured the associations between the two items regarding TFWPs and resource availability for and valuing of TRTTF implementation, comparing results from medical healthcare centers (MHCs) and substance use treatment centers (SUTCs), while this current work measured the association between these two items and provider attitudes toward concurrent treatment of tobacco and substance use. The research aims guiding each study are unique. Additionally, comparative analyses were not conducted between MHCs and SUTCs in this study.

Other publications related to the TRTTF project include Siddiqi et al. [23], a quantitative analysis of differences in provider beliefs and delivery of a brief tobacco cessation intervention for cigarette and non-cigarette tobacco use, the 5A’s, comparing results between the same 10 SUTCs and 9 MHCs from the same sample of 347 providers. The same center characteristics are reported, and the same response-rate figures, including use of the Mann–Whitney results for unique patients. Wani et al. [56] presented a qualitative study on 6 early adopters of TRTTF and shared 6 interviews out of the 12 analyzed in the current study. Rogova et al. [57] conducted a qualitative case study on 1 center participating in the TRTTF program and shared 1 interview with the current study. Feyisetan et al. [58] performed a quantitative study, and the primary variables of interest included employee tobacco-related knowledge and receipt of tobacco-related training by TRTTF within 8 SUTCs and 8 MHCs, and these 16 HCs overlap with the 19 in the current study. As the sample for the Feyisetan study was 16 centers rather than the same 19 centers, different sample characteristics are reported.

### 2.3. Procedures and Participants

#### 2.3.1. Eligibility and Recruitment

This manuscript presents findings from a convenience sample of a statewide initiative aimed at helping healthcare and community organizations implement TFWPs and practices in rural and MUAs of Texas. Originally launched in 2013, Taking Texas Tobacco-Free (TTTF) has evolved through multiple funded grants to address the unique needs of diverse healthcare systems across Texas. The current evolution, Taking “Rural” Texas Tobacco-Free (TRTTF), focuses on engaging two types of HCs: MHCs and SUTCs that serve rural communities and/or medically underserved areas (MUAs). TRTTF is a comprehensive program that seeks changes on the level of the organization to build system-wide, sustainable initiatives that aim to transform organizational culture and practices regarding treating tobacco dependence among community healthcare partners serving rural/MUAs. Participation was open to centers whose leadership self-identified as meeting the geographic criteria and expressed interest in strengthening their tobacco control efforts.

To identify eligible organizations, the project team compiled a statewide list of HCs operating in rural areas and/or MUAs. This list was created primarily using online research, directories from professional organizations, and past partnerships. Outreach efforts were conducted primarily via email and included a flyer outlining the goals and offerings of the TRTTF initiative. Additional recruitment took place through engagement at professional events relevant to rural healthcare providers, such as conferences hosted by the Texas Association of Rural Health Clinics, the Texas Association of Community Health Centers, and the Texas Association of Addiction Professionals. At these events, the program was promoted through booth displays, flyer distribution, and/or presentations. Interested organizations were invited to provide contact information or complete a brief online survey to confirm eligibility.

#### 2.3.2. Study Procedures

Our data collection period spanned from August 2021 to April 2025, a period that overlapped significantly with the COVID-19 pandemic, which slowed data collection due to center closures, staff shortages, and center attrition over time. Nineteen centers (9 MHCs and 10 SUTCs) enrolled in the program by signing a Memorandum of Understanding with the project team. Centers were enrolled on a rolling or continuous basis, and the projected program implementation timeline was 1–1.5 years for each center. Prior to implementation, staff at each center were invited to complete a baseline survey designed to assess organizational characteristics and readiness. The survey was distributed electronically via Qualtrics (https://www.qualtrics.com), with center leadership or a designated program champion, a volunteer manager or provider facilitating internal dissemination, who was not financially compensated for this position. Respondents were asked to indicate their role as managers or direct service providers, with the latter defined as individuals holding clinical or support credentials (e.g., nurse practitioners, medical assistants, licensed chemical dependency counselors, and peer support specialists). To encourage participation, providers were offered a USD 10 Amazon e-gift card, contingent on employer approval. Gift card information was collected separately to ensure anonymity—no other identifying data were gathered.

Data were collected from centers within 5 months of enrollment with 2 exceptions. In both cases, scheduling of group interviews was delayed by our program partners. Many of the HCs partnering with us in this project labor under many organizational challenges, including being under-resourced regarding funding, staff, and servicing a high volume of patients. These 2 centers experienced difficulties in pulling staff out of service to participate in the group interviews. The interval between program enrollment and completion of the group interview in one center was 8 months, and 13 months in the second center. However, both of these centers were delayed in completion of pre-implementation activities, such that the late completion of the staff group interview was still within the pre-implementation phase. The center with the 13-month delay oversaw 6 individual clinics and had experienced an organizational restructuring, high staff turnover, and departure of the program champion. Thus, at the time of the pre-implementation group interview this center was still in the pre-implementation phase, patients were not being provided any tobacco-free services, not even referral to Texas Tobacco Quitline, as participants were unaware of the Quitline, the center had not yet received NRT, their tobacco-free signage, or educational materials. Most importantly, however, this center had not yet scheduled a key component of the program, periodic check-in meetings with our implementation specialists to provide them with hands-on guidance through the implementation process tailored to their individual needs. As each center was unique, there were variations in the timespan for completing pre-implementation data collection. The shortest interval between enrollment and data collection from surveys and interviews was 1.5 months, and the longest was 13 months, as noted above.

Different types of purposive sampling strategies, criterion as well as total sampling, were used for selection of the qualitative sample. Individual or group interview participants were recruited according to criterion sampling, in which participants are purposively selected based on predetermined criteria [59]. Criteria for participants included individuals who provided direct services to clients, and managers—individuals who were involved in program implementation in some way. Total sampling was also used, which entailed selecting the entire population that fit our sample criteria [60]. Selection of interview participants was coordinated through program champions. Program team members consulted program champions at each center to enlist their assistance in coordinating and scheduling interviews in accordance with preset criteria. Program champions were center clinical or management staff who volunteered to oversee program implementation without additional financial compensation. Emails to program champions describing interview procedures and particulars included the following text, explicitly stating that group interviews should not include supervisors and those they supervise: “We ask that supervisors and those they directly supervise not be included in the same group interview, to help staff feel comfortable and speak openly about their experiences and views.” Individual interviews with supervisors were arranged to accommodate this concern.

#### 2.3.3. Quantitative Sample

As commonly reported in mixed-methods designs, the qualitative and quantitative samples differ in this study. A total of 542 staff members from 9 MHCs and 490 staff from 10 SUTCs completed the pre-implementation anonymous survey, corresponding to average response rates of 73.61% and 74.63%, respectively, based on staff census figures provided by leadership. For analysis, the sample was narrowed to include only direct service providers, 173 from MHCs and 174 from SUTCs, as these individuals were the only ones asked about provider attitudes toward concurrent treatment of tobacco use and BHCs and their valuation of tobacco use services. Within this subset, the average response rates were 49.19% for MHC providers and 49.45% for those from SUTCs. Across all participating MHCs and SUTCs, the overall provider response rate was 29.56% (347 responding providers out of 1174 total service providers).

#### 2.3.4. Qualitative Sample

The qualitative sample was drawn from 12 HCs, comprising 7 SUTCs and 5 MHCs, that also contributed to the quantitative dataset. In total, 85 individuals participated in 16 virtual semi-structured individual (*n* = 2) and group (*n* = 14) interviews conducted prior to program implementation, including 70 direct service providers and 15 managers, across both individual and group formats. The direct service providers included the following titles and credentials: licensed professional counselor (*n* = 10); licensed chemical dependency counselor (*n* = 6); community outreach specialists (3); healthcare/patient navigator (*n* = 5), nurse practitioner (*n* = 3); licensed vocational nurse (*n* = 4); certified medical assistants (9); youth prevention counselor (*n* = 1); tobacco treatment specialist (*n* = 1); peer support specialist (*n* = 6); intellectual and developmental counselor (*n* = 3); adult mental health counselor (*n* = 4); registered nurse (*n* = 6); recovery coach (*n* = 3); case manager (*n* = 6). Managers who did not provide client direct services included the following titles: clinical manager (*n* = 7); human resource director (*n* = 2); information technology manager (*n* = 2); program director (*n* = 4). Pre-implementation interviews were generally completed 5 months after enrollment, following initial planning and preparation activities (e.g., obtaining institutional approvals, developing initial scale-up plans, etc.). Unlike the quantitative sample, managers were included in the qualitative sample, because they could provide valuable insights into staff leadership and organizational attitudes toward concurrent treatment of tobacco and substance use within their center.

### 2.4. Quantitative Measures

#### 2.4.1. Provider Attitudes Toward Concurrent Treatment of Tobacco Use and Behavioral Health

Respondents reported their attitudes toward concurrent tobacco use and behavioral health treatment by rating their agreement with one survey item on a 5-point Likert-type scale ranging from 1 = strongly disagree to 5 = strongly agree. To analyze the data, consistent with our prior work [53], responses were dichotomized into two categories: “yes” (combining strongly agree and somewhat agree) and “no” (combining neither agree nor disagree, somewhat disagree, and strongly disagree). This item was developed by the research team for the purpose of the multistage evaluation project. It assessed whether providers believed that people with mental health and non-nicotine SUDs who quit smoking may experience concurrent improvements in mental health symptoms and/or reductions in non-nicotine substance use.

Respondents reported their attitudes toward concurrent tobacco use and non-nicotine SUD treatment by selecting one of three responses to a single-choice question asking for their opinion on the sequence of tobacco and illicit drug cessation: “Quit using drugs,” “Quit using tobacco,” or “Quit using drugs and tobacco at the same time.” As the present study’s focus was on attitudes toward concurrent treatment, responses were dichotomized into two categories: 1 = “Quit using drugs and tobacco at the same time,” and 0 = “Quit using drugs” or “Quit using tobacco.” This item was also developed by the research team for the purpose of the multistage evaluation project. It assessed whether providers believed that people with non-nicotine SUDs should quit using tobacco products and illicit drugs concurrently.

#### 2.4.2. Providers Change Value Regarding TFWPs

Respondents were asked to rate their agreement with statements assessing their change value regarding TFWPs on a 5-point Likert-type scale ranging from 1 = strongly disagree to 5 = strongly agree. The statements assessed whether TFWPs are important because they provide a clean and safe environment for staff and patients, as well as help patients and staff quit smoking. Following our previous work [53], responses were dichotomized into two groups: “yes” (strongly agree or somewhat agree) and “no” (neither agree nor disagree, somewhat disagree, or strongly disagree).

#### 2.4.3. Descriptive Statistics

Respondents reported on the following center-level characteristics: (1) the number of counties served, (2) the number of unique annual patients treated, and (3) whether the center had an existing comprehensive TFWP. At the provider level, respondents who identified as providers reported whether they received training on concurrent treatment of tobacco and non-nicotine substance use within the past year.

### 2.5. Qualitative Data Collection

Research aims guided all qualitative data procedures, including sampling strategies, writing up interview guides, as well as selection of data collection and analytic methods [59]. Research questions and instruments were developed by a trained qualitative researcher, I.M.L., a cultural anthropologist and public health researcher based on previous experience in implementing tobacco control interventions within HCs serving under-resourced or marginalized groups. Aims explored staffs’ (i.e., direct service providers and managers) perspectives, experiences, and attitudes around addressing tobacco dependence by adopting a TFWP, screening for, and treating tobacco dependence in general, and specifically in relation to (1) provision of concurrent treatment of tobacco and behavioral health needs, and (2) their valuing of implementing TFWPs within their HCs. These data were collected as part of a formative evaluation process aimed at informing and expanding these HCs’ capacity for successful program implementation.

Members of the primary implementation research team, our health education specialists, engaged in participation observations during initial site visits at participating centers, writing up summaries of their observations that were shared with the team. These summaries were analyzed by the qualitative analysts and helped inform the development of the pre-implementation semi-structured interview guide. The findings from these site visits are beyond the scope of this manuscript but are described here to provide broader context for the study’s exploratory sequential design. A semi-structured interview guide based on research aims, prior research experience, and analysis of participant observation data was used that was field-tested and revised according to responses in the field and reviewed by study team members. Individual or group interviews were either audio and video recorded (online) or audio recorded (in-person) and transcribed by a professional HIPAA-compliant transcription company. Group interviews generally consisted of 4–10 participants. Interviews were conducted by I.M.L., lasting ~30 to 60 min, varying according to number of participants. Although this study is considered a quality improvement project, which does not meet the regulatory definition of human subjects research, best research practices were applied for qualitative and quantitative procedures, including informed consent processes. Interview participants were informed of the study specifics and the voluntary nature of their participation and confidentiality and privacy procedures. Oral consent was obtained from all participants prior to study participation. Pre-implementation interview questions focused on understanding centers’ current TFWPs, practices, attitudes toward, as well as barriers and facilitators to, program implementation, with the aim of guiding program adaptation. Transcripts were checked for accuracy against the recordings and corrected prior to ensuring de-identification and destruction of recordings. Participants were compensated with a USD 20 electronic gift card for their participation.

### 2.6. Data Analysis

#### 2.6.1. Quantitative

Descriptive statistics summarized key study variables. Provider survey responses were summed to calculate both absolute and relative frequencies regarding provider attitudes toward concurrent treatment and the value of tobacco-free workplace programs, including policies and cessation practices. Generalized linear mixed models (residual pseudo-likelihood estimation method, binomial distribution, logit link, and variance components for the variance matrix) analyzed the relationships between attitudes toward concurrent treatment and outcomes of interest, perceived program value, adjusting for whether the health center had an existing TFWP (*n* = 10) and providers’ past-year receipt of training on concurrent treatment of tobacco and non-nicotine substance use (*n* = 45). The final sample consisted of 347 providers from 19 HCs spread across rural areas and MUAs of Texas. Because the analyses were restricted to completed pre-implementation survey responses, there were no missing data to account for in the analytic sample. Given the restricted geographic areas in one state, this study serves as an exploratory purpose, with a more generous significant level of *p* < 0.05, despite conducting four separate statistical analyses. All analyses were conducted using SAS version 9.4.

#### 2.6.2. Qualitative

A rapid qualitative analysis (RQA) approach was used based on Hamilton’s [61] work and matrix analysis [62]. This analytic approach was selected specifically, as it aligned with our research aims to quickly and rigorously assess community partners’ needs and to inform adaptation of implementation plans and interventions to local contexts. Templates were created of key domains that were drawn directly from interview guides, with each domain corresponding to an interview question, and an additional domain for data that did not correspond to any existing domains, thus allowing for the adaptation and refinement of the template. Two team members trained in RQA (I.M.L. and T.D.G.) initially used the template to summarize the same five transcripts and confirm the reliability and appropriateness of the domains, i.e., that the domains could be identified in the data, as well as establish consistency in summarizing across team members [63]. Links to the original transcripts were included in all RQA summaries for easy access to the raw data. Once consistency had been established, the transcripts were divided between team members for summarizing using the template. Summaries took the form of 2–3-page concise bulleted points that also included significant quotes to exemplify key issues. Team analysts met regularly to compare and discuss summaries to reach agreement and to collaborate on the development of a matrix that was informed by research questions and based on domain constructs. Bulleted summaries and quotes were then transferred to the matrix to allow for systematic comparison across participants’ responses. Final analysis entailed the first (T.D.G., a graduate student in counseling psychology) and second authors (I.M.L.) meeting to conduct matrix and thematic analysis, systematically comparing data across the matrix to synthesize data further. This was an inductive, thematic analysis process that relied on constant comparison to compare and synthesize data across sites to develop final themes [64].

Upon completion, the RQA summaries were shared with the implementation team, in this case, health education specialists, the primary liaisons between the larger study team and community partners. Health education specialists reviewed and converted findings into action items, focused on practical applications to address partners’ concerns and facilitate the tailoring of interventions to local contexts. These initial action items were then discussed at bi-weekly meetings with the study team for further refinement and were added to agendas for monitoring of task completion. Finally, health education specialists discussed with program champions the feasibility and possible next steps for implementing any recommended changes to support program implementation within their center. Methodological procedures for RQA in this study were guided by Kowalski et al.’s framework to enhance analytic rigor [65]. The entire process from collecting, transcribing, writing up RQA summaries, matrix, and thematic analysis, writing up action items, and sharing them with the study team in meetings generally lasted 3 months.

## 3. Results

### 3.1. Quantitative Results

#### Healthcare Center Characteristics

Table 1 summarizes the descriptive characteristics of the participating MHCs and SUTCs, as reported by their leadership, including the number of counties served, unique patients served annually, total annual patient visits, number of direct service providers, and whether a TFWP was already in place. There were no statistically significant differences between MHCs and SUTCs in select characteristics.

As high provider tobacco use rates have been recognized as a barrier to TFWP implementation, providers were surveyed on their tobacco use. Results from Table 2 show provider tobacco use rates of 7.78% for combustible cigarettes, 9.51% for electronic cigarettes, and 6.05% for alternative tobacco products (e.g., Snus, hookah, dissolvable tobacco products, and chewing or dip), with 92.22% endorsing not smoking or having quit completely.

Results showed that, compared to providers who did not hold these attitudes, those who agreed that concurrent treatment improves BHCs were more likely to also agree that TFWPs promote clean, safe environments (OR = 3.81; 95% CI: 2.31–6.28; *p* < 0.001) and help staff and patients quit smoking (OR = 3.04; 95% CI: 1.90–4.87; *p* < 0.001). Similarly, providers who supported the idea that individuals with non-nicotine SUDs should quit tobacco and other drugs at the same time were more likely to agree that TFWPs aid smoking cessation among staff and patients (OR = 1.77; 95% CI: 1.08–2.92; *p* = 0.024). Lastly, the low intraclass correlation coefficient values suggested minimal variation between centers. Model results are presented in Table 3, Table 4 and Table S1.

### 3.2. Qualitative Results

Analysis of interview transcripts revealed three themes regarding HC staff members’ (i.e., providers and managers) support and practices for addressing tobacco dependence among patients with BHCs, as well as attitudinal barriers affecting tobacco-free workplace program implementation, further delineated into constitutive subthemes (Table 5): (1) prioritization of “harm reduction” techniques, (2) limited support for providing tobacco use screenings and tobacco cessation services, and (3) hesitation about tobacco-free workplace program integration. Each theme is described below, accompanied by direct quotes from the study participants.

#### 3.2.1. Prioritization of “Harm Reduction” Techniques

Participants expressed concerns about implementing TFWPs that could potentially harm their patients and expressed concern for their other BHC treatment, citing patients’ use of tobacco products as a way to help manage and cope with their other BHC treatment. One expressed:

*One of my clients is—she’s dealing with a severe eating disorder. So, cigarettes go hand in hand with that for her. So, when she has reported trying to stop in the past, her eating disorder became a lot worse, and she became a lot more suicidal. So, with her particularly, her stopping cigarettes would be a major concern because she is struggling with maintaining her eating disorder and her suicidal ideas. So, cigarettes kind of help calm her, and balance her currently.*

(SUTC #6)

Additionally, they indicated that smoking cigarettes was considered a “better” option compared to the other substances their patients used and indicated they may not be capable of concurrently quitting tobacco and illicit drugs. Participants’ particular misunderstanding of the concept of “harm reduction” was one that focused on allowing patients to use less harmful substances, i.e., tobacco as opposed to illegal ones, because it is less harmful and less stigmatizing than the negative consequences associated with drug use:

*So, in implementing a tobacco-free campus, doing so in a way where we are still reducing stigma and harm, especially when working with the clientele we serve. So, for them, if they’re smoking instead of using IV heroin, then to them, that’s a better choice.*

(SUTC#4)

Moreover, participants expressed concerns about patients becoming frustrated by tobacco-free program implementation while receiving acute care. The suggestion being that not being able to use tobacco while recovering from other substances would cause more harm rather than reducing it, and thus not aligned with “harm reduction.” One recalled:

*I can say, at a past job, I used to work in a small inpatient unit for postcrisis, people coming out of the hospital and stuff, and they had a very strict no-smoking policy, and they would force the clients to switch to gum or patches or essentially, they wouldn’t be able to stay there. That didn’t really go well almost ever, people would be really upset. They’d be like, “I’m coming off of a serious overdose, and you won’t even let me smoke. How do you expect me to recover from that? You’re taking away everything,” like almost never did that go well.*

(SUTC #3)

Some participants stated that patients should initiate tobacco cessation discussions and express the desire to quit before providers offer resources or interventions, in accordance with providers’ conception of harm reduction as allowing patients to choose their treatment, given that patients are contending with various issues, some of which they prioritize over tobacco cessation. One participant said:

*“I think that we have to prioritize what our clients want us to prioritize. So, if they see their number one problem is their substance use is, say, heroin, then that’s the one that we’re going to focus the most on. If they think that their main goal is to stop smoking, then that’s the one we’re going to focus on… We allow the client to make the choice on the way that their treatment and recovery goals would be.”*

(SUTC#7)

#### 3.2.2. Limited Support for Providing Tobacco Use Screening and Tobacco Cessation Services

Many participants said they had not received education or training on tobacco use treatment but showed interest in learning about it. One participant said, “*So, once we get educated, we get our flyers, we get our NRTs from you, we can start moving with that process as part of our program*” (SUTC #6). Additionally, some participants reported that their health centers lacked the necessary resources to offer tobacco use counseling. One participant explained, “*We really didn’t have maybe go-to solutions to offer patients, such as the NRT, the Quitline, things of that nature. We didn’t have anything. We would just–”Oh, stop smoking,” you know?*” (MHC #5).

Moreover, participants from MHC #1 indicated that they were unaware of the Texas Tobacco Quitline when asked about their tobacco use treatment and referral practices during their interview. Other participants reported underutilization of the Texas Tobacco Quitline due to concerns about providing unwanted interventions for patient tobacco use. One stated:

*Maybe if there’s like an alternative that I can tell them, “There’s a number you can call to speak about it whenever you decide if you want to stop,” because sometimes, they don’t want to talk about it in person, because I guess they feel like maybe it’s like an intervention almost.*

(MHC #5)

While another explained further that there may be differences in how patients receive the referral information when it is provided by staff and when it is discussed with medical providers. They explained:

*I feel like they’d be more receptive where it’s coming from the* [medical] *provider than us. Us, they kind of would close off a little bit because we’re not the* [medical] *provider, but trying to somehow integrate, encourage them to attend that training so that they can help us reinforce the importance, because they know their* [medical] *providers.*

(MHC #5)

Others mentioned that they did not have the organizational resources, i.e., generally they did not have enough time to address tobacco cessation during patient visits. One remarked, “*Well, honestly, I’m too busy. I’m going to address what they’re there for. Those extra things that I don’t hit on them.*” (MHC #2)

#### 3.2.3. Hesitation About Tobacco-Free Workplace Program Integration

Several participants expressed doubt about their patients’ willingness to quit, noting that patients use tobacco to cope with other stressors. They also highlighted that tobacco use is deeply ingrained in their healthcare settings. One participant explained:

*The reality is a lot of our vets aren’t going to quit smoking, and it’s because it’s too much of an ingrained thing, and there are very few, I don’t know, coping mechanisms that are as readily available and accepted as smoking.*

(SUTC #3)

Participants further expressed concerns about forcing patients to make changes that they are not ready for, potentially encroaching on their bodily autonomy. One stated:

*… We have to be cautious of this autonomy, this whole sense of like I—especially if they have a dual substance use disorder, usually they compensate the one with the other. So, they quit a hard drug, then they’re smoking, so I can see that a little bit, but for the most part, I mean this is something that we would offer them. It’s not something we’d force them to do.*

(SUTC #3)

Additionally, participants questioned their patients’ ability to remain abstinent from tobacco after completing their programs. They voiced concerns about patients’ access to resources like NRT:

*Maybe once the clients complete their 90 days, that might be an issue because they might still want to come back for NRT replacement therapy. It might be in competition with those that are currently in the program. So, it might not have no supplies to continue to help them on.*

(SUTC #1)

## 4. Discussion

Despite well-established guidelines recommending universal screening and behavioral intervention delivery for those using tobacco [22], evidence-based tobacco treatment services remain underutilized in HCs generally, and those treating BHCs in MUAs particularly. As provider buy-in can heavily influence tobacco intervention delivery, this study utilized a mixed-methods approach to examine healthcare provider attitudes on concurrent treatment of tobacco and BHCs, as well as the perceived value of TFWPs in HCs serving patients in rural areas/MUAs of Texas. Further, we explored qualitative themes and subthemes underlying these provider attitudes and values to inform future tobacco treatment delivery and successful tobacco-free workplace program implementation in rural/MUAs of Texas. Quantitative findings showed that providers who agreed that concurrent treatment improves BHC outcomes were more likely to agree that TFWPs help staff and patients quit smoking and that TFWPs create a clean and safe environment. Providers who endorsed concurrent quitting for people with non-nicotine SUDs were more likely to agree that TFWPs are helpful for cessation among staff and patients. However, this belief did not extend to the agreement that TFWPs provide a clean and safe environment. Qualitative findings revealed three core themes highlighting minimal organizational support and practices for addressing tobacco dependence among patients and attitudinal barriers: (1) prioritization of “harm reduction” techniques, (2) limited support for providing tobacco use screenings and tobacco cessation services, and (3) hesitance regarding tobacco-free workplace program integration. These themes contextualize and offer an explanation for quantitative results, revealing the factors underlying provider attitudes toward concurrent treatment. With tobacco use remaining the leading preventable cause of death and disproportionately affecting people with BHCs in the U.S. [4], and disproportionately affecting those in rural areas and MUAs who report higher smoking rates and poorer health outcomes [38,39], provider experiences and perspectives must be understood so that appropriate efforts for education, training, and resource provision to support tobacco intervention efforts can be made in settings where these disparities are most pervasive.

A primary finding of the present study was that providers who perceived concurrent treatment as helpful for improving BHCs were more likely to endorse that TFWPs help staff and patients quit smoking. However, there was low support for concurrent treatment, with only 32.85% of providers endorsing that people with non-nicotine SUDs should quit using other drugs/concurrently. Likewise, qualitative findings suggest that providers’ and managers’ attitudes toward concurrent treatment may also function as barriers to TFWP implementation and tobacco dependence treatment delivery. Integrating qualitative findings with quantitative results helps explain the basis for this low support for concurrent treatment by providers across multiple locations based on their concerns about patients’ willingness and ability to undergo concurrent treatment. Providers also frequently reported that it was common practice for patients to engage in tobacco use to cope with other drug-related treatment. Many providers also reported using a harm reduction approach when treating patients. Providers’ understanding of harm reduction reflected a patient-led prioritization of care, in which tobacco dependence treatment was often not prioritized unless the patient explicitly expressed a need or desire for it. While respecting patient autonomy is one of the six principles of harm reduction, this model focuses on supporting patients in reducing unhealthy behaviors in a humane, pragmatic, and individualized, incremental manner, rather than disregarding these adverse health behaviors [66]. The sixth principle of harm reduction is known as “accountability without termination,” which entails assisting patients in understanding that they are responsible for their health behaviors and choices as well as the consequences of those choices [66]. As noted by Prochaska (2010) [30], the failure to treat tobacco use within mental health and substance use treatment settings has been rationalized as a clinical approach to “harm reduction.” This rationalization is based on the idea that tobacco use is less harmful than alcohol or illegal substance use, a misconception, as more than 53% of individuals treated for substance use disorders die from tobacco-related diseases rather than the substance for which they seek treatment [32]. The theme prioritization of “harm reduction” techniques presented participants’ misunderstandings regarding the harm reduction model, and the rationalizations they gave for not addressing client tobacco use. These attitudes revealed by the qualitative data serve to explain the low to moderate provider quantitative results regarding the value of concurrent treatment. Integrated findings suggest that providers’ perception and valuation of concurrent treatment is based on a misconception that it could jeopardize patient BHC recovery and deprive them of a valuable and less “harmful” means of coping with the stress of recovery. Under these circumstances, providers would be justified in not supporting a treatment that they judged to be harmful to their patients. These findings echo prior research that revealed a pervasive belief among healthcare providers that patients engage in tobacco use to cope and are often not interested in quitting, impacting their approach to concurrent treatment [28]. Moreover, the present findings also expand on prior research highlighting that, despite knowledge of clinical guidelines, healthcare providers reported taking a hierarchical approach to tobacco dependence treatment in which those with active tobacco use health conditions and more severe use are more likely to be encouraged to engage in tobacco dependence treatment than those who are more infrequent tobacco users [67]. Consistent with the earlier findings, participants in this study noted that their tobacco use practices are influenced by their knowledge on treating tobacco use and expressed a desire for additional training [67]. In rural areas and MUAs, these educational and training opportunities may be especially helpful in countering social norms surrounding tobacco use, which are bolstered by historical tobacco farming practices that have shaped community attitudes toward tobacco use that differ from those in urban settings [68].

Providers who agreed that concurrent treatment improved BHC outcomes were also more likely to agree that TFWPs provide a clean/safe environment. However, qualitative results highlighted persistent concerns regarding TFWP implementation, including fears that TFWPs may stigmatize patients and skepticism about patients’ ability to sustain abstinence from tobacco use after substance use treatment program completion. While NRT can be acquired online, at convenience stores, and through the Texas Tobacco Quitline, providers expressed concerns about patients’ potential loss of access to NRT from the treatment center. Notably, these concerns reported in the qualitative data may reflect a knowledge gap among providers regarding tobacco treatment resources, which may contribute to provider hesitation to treat tobacco use, gaps in the continuity of care for patients with BHC needs, and challenge successful TFWP implementation. Only a modest percentage (63.68%) of providers agreed that concurrent treatment improves BHCs, which qualitative data reported as related to concerns about patients needing to use tobacco to cope with stress and inability to maintain reduction or cessation after leaving treatment and losing access to NRT, as noted above. Similar to earlier findings, which indicated that providers were more supportive of tobacco cessation treatment and training than of TFWPs in inpatient and outpatient mental health treatment centers, the present findings highlight mixed attitudes among providers toward tobacco cessation treatment and policies [69]. These mixed attitudes and resulting gaps in continuity of care may be particularly consequential in rural areas and MUAs, where patients may lack alternative care options. Collectively, these mixed attitudes toward tobacco cessation interventions and policies underline a need for education and training on comprehensive tobacco-free workplace program implementation encompassing both policy adoption and cessation support that emphasize non-judgmental, patient-centered approaches, sustained access to evidence-based treatments, and highlight positive impacts on holistic health for HC staff and patients.

Providers in the present study who endorsed concurrent quitting for people with non-nicotine SUDs were more likely to perceive TFWPs as effective for supportive tobacco cessation among staff and patients. However, this endorsement did not extend to the agreement that TFWPs provide a clean and safe environment, suggesting selective valuation of tobacco control intervention efforts. Combining this result with the qualitative findings suggests that providers worry TFWPs may be perceived as coercive or as infringing on patients’ autonomy, thereby contributing to an unsafe environment. This may explain why this attitude was not associated with endorsing the statement that TFWPs contribute to clean and safe environments, even though providers generally agreed with that statement. This pattern may reflect an underlying disconnect between provider support for tobacco cessation as an individual-level clinical intervention, as opposed to environmental or institutional-level structural strategies. Similar distinctions have been observed in prior research, including qualitative findings from substance use treatment settings highlighting providers’ hesitance toward TFWP implementation due to concerns about patients potentially pushing back and choosing different treatment centers that were not tobacco-free [70]. Additional evidence from the Los Angeles County Health Agency suggests that while providers broadly supported smoking cessation interventions, mental health providers specifically were less likely to agree that tobacco use identification was a necessary part of their job responsibilities, further demonstrating fragmentation in role expectations across various care settings [71]. Together, these findings further demonstrate the need for comprehensive tobacco-free workplace programs that include education, training, communication, and sustained resource allocation to reduce tobacco use and increase provider support for the adoption of TFWPs in HCs, as emphasized by the Centers for Disease Control and Prevention [18,72]. Further, for HCs serving rural areas and MUAs with smaller operating budgets, the findings underscore the vital need for adequate, low-cost resources to support program implementation and build organizational capacity, including the Texas Tobacco Quitline, NRT distribution programs, and tailored assistance programs, such as the TRTTF initiative.

While the mixed-methods study design and the novelty of the topic demonstrate the study’s key strengths, a few limitations should be considered. First, the present study has a cross-sectional design, which precludes causal inferences regarding the relationships between provider attitudes, concurrent treatment endorsement, and TFWP implementation. Although the findings provide valuable insight into provider attitudes prior to tobacco-free workplace program implementation, future research may benefit from utilizing longitudinal study designs to examine causal pathways. Second, as this study focused on attitudes toward TFWPs and concurrent treatment in areas with limited access to care (i.e., rural areas and MUAs) and occurred amid broader institutional changes driven by rapidly evolving health needs (i.e., COVID-19), the findings may not be generalizable to other similar settings. Although COVID-19 may have affected generalizability, it also brought widespread changes to healthcare delivery, particularly the introduction of telehealth services. Initially used to help facilities remain operational while adhering to social distancing guidelines, telehealth has persisted and now also increases access to care, especially in rural and MUAs. As such, the present study’s findings may still provide valuable insight into barriers to TFWP implementation and concurrent treatment delivery in rural and MUAs. Third, provider responses to the report measures and interviews may have been influenced by social desirability bias, especially given their HC’s participation in a broader tobacco-free workplace program implementation initiative. However, the mixed-methods study design may have helped partially mitigate this limitation. Although survey responses were not directly linked to individual interview participants, the integration of quantitative and qualitative results focused on contextualizing quantitative responses to infer organizational readiness rather than directly explaining them at the individual level. Still, because this study used anonymous surveys and focused primarily on how provider attitudes influence organizational culture and practices regarding tobacco interventions, we did not collect individual demographic information, limiting our ability to compare respondents with the broader workforce or assess additional provider-level characteristics that may affect the identified associations. Additionally, because the survey items were developed by the research team for the multistage evaluation study and have not undergone psychometric validation, the findings should be interpreted as indicators of provider attitudes within the context of this program. Moreover, both program enthusiasts and detractors were purposively recruited for interviews to mitigate social desirability bias. Additionally, to support participant comfort in openly sharing their perspectives during pre-implementation group interviews, direct supervisors were not interviewed in the same group with their supervisees. However, the convenience nature of the sample consisting of HCs that agreed to participate in and did not drop out of the Taking Texas Tobacco-Free program limits the generalizability of the findings. Finally, the study’s geographic focus on providers working in HCs in rural areas and MUAs limits the generalizability of this study’s findings to other settings. Still, this focus enhances the relevance of the findings for HCs serving underserved and historically understudied populations, where implementation barriers and provider attitudes may differ significantly from those in higher-resource settings. Thus, the present study’s findings may help advance knowledge and improve implementation efforts in settings with limited evidence and significant public health need. Future studies may benefit from examining provider attitudes toward concurrent treatment and TFWPs in varied settings to inform future individual-level clinical interventions and environmental- or institutional-level tobacco control strategies.

## 5. Conclusions

Integration of qualitative and quantitative results identified three main findings: (1) ambivalent support for concurrent treatment amid limited organizational resources, (2) concerns about concurrent treatment jeopardizing BHC recovery, and (3) mixed feelings about TFWP implementation, especially regarding patient autonomy, stigma, and feasibility. These findings expand existing literature by highlighting that provider support for concurrent tobacco and behavioral health treatment does not necessarily translate into support for TFWP implementation. This underscores the need to address both provider-level attitudinal and structural barriers to tobacco control efforts, such as tobacco-free workplace programs, inclusive of TFWPs. The mixed-methods approach provided a more nuanced understanding of how factors such as provider attitudes, organizational practices, and resource limitations affect tobacco treatment delivery in health centers, particularly in rural areas and MUAs. Notably, provider attitudes may reflect adaptations to systemically and structurally under-resourced environments, where treatment options must account for patients’ primary concerns, varying social norms surrounding tobacco use, staff shortages, limited tobacco-related education and training opportunities, and limited alternative care options for patients. Future efforts to overcome these barriers may benefit from comprehensive tobacco-free workplace programs that incorporate TFWPs, provider education, tobacco control training, and sustained resource provision to boost organizational capacity. Addressing these structural supports may improve tobacco treatment for patients with behavioral health needs and help reduce tobacco-related disparities in rural and MUA settings.

## Figures and Tables

**Table 1:** Select leadership-reported pre-implementation characteristics of medical healthcare centers (MHCs) or substance use treatment centers (SUTCs) serving rural and/or medically underserved areas in Texas, U.S., agreeing to participate in a comprehensive tobacco-free workplace program (*N* = 19).

Select Center Characteristics	All Centers (*N* = 19)		MHC (*n* = 9)		SUTC (*n* = 10)		MHCs vs. SUTCs	
**Number of:**	**Mean** **(SD)**	**Median** **(IQR)**	**Mean (SD)**	**Median (IQR)**	**Mean (SD)**	**Median (IQR)**	**Statistic**	***p*** **-Value**
Counties served (*n* = 19)	7.89 (11.51)	3 (1–7)	2.56 (1.67)	2 (1–3)	12.70 (14.43)	7 (1.25–7)	−1.4143	0.1743
Unique patients/yearly (*n* = 18)	6094.56 (10,688.54)	2090.5 (1000–6826)	6102.89 (4255.18)	6500 (3000–10,239)	6086.22 (14,988.79)	1202 (960–1500)	−2.0772	0.0532
Total patient visits/yearly (*n* = 18)	21,470.89 (28,909.46)	10,700 (1800–25,000)	20,127.78 (11,386.81)	22,000 (14,400–25,000)	22,814.00 (40,524.87)	3000 (1086–6000)	−1.5903	0.1302
Direct service providers (*n* = 19)	61.79 (83.66)	25 (10–75)	58.67 (52.75)	58 (15–71)	64.60 (107.27)	14.5 (7–75)	0.8169	0.4247
	**%**	***n***	**%**	***n***	**%**	***n***		
Extant TFWPs	52.63	10	44.44	4	60.00	6	0.4598	0.6563

Note. Mann–Whitney tests were conducted to examine differences by center type. TFWPs = tobacco-free workplace policies; IQR = interquartile range.

**Table 2:** Types and frequency of provider tobacco use in Texas healthcare centers serving rural and/or medically underserved areas (*N* = 347).

Item	% [*n*]/Mean (SD)
**Full-time employee** †	82.13 [285]
**How often do you smoke cigarettes?**	
Every day Some days Weekends Only when I drink alcohol I do not smoke or have quit completely	4.03 [14] 0.86 [3] 0.29 [1] 2.59 [9] 92.22 [320]
**On how many of the past 30 days did you smoke cigarettes?**	17.15 (13.49)
**On the days that you smoke how many cigarettes do you smoke each day?**	6.19 (4.95)
**Have you used any of the following other/alternative tobacco products more than 1 time within the last year?** Snus, such as Camel or Marlboro Snus Roll your own cigarettes Tobacco from a hookah or a waterpipe Dissolvable tobacco products like Ariva/Stonewall/Camel/Camel Orbs/Camel sticks Electronic cigarettes or E-cigarettes (including battery-operated vape pens, e-pipes, and e-cig) Cigars Little cigars/cigarillos/bidi’s Chewing tobacco, dip, or snuff Other tobacco products (besides conventional cigarettes, please specify)	1.15 [4] 0.00 [0] 1.15 [4] 0.00 [0] 9.51 [33] 1.44 [5] 0.58 [2] 1.44 [5] 0.29 [1]

Note. † Missing information for some respondents for this value.

**Table 3:** Providers’ attitudes on concurrent treatment of tobacco use and behavioral health conditions, perceived value of tobacco-free workplace policies in Texas healthcare centers serving rural and/or medically underserved areas, and receipt of past-year trainings on concurrent treatment (*N* = 347).

Item	% [*n*]
**Provider attitudes toward concurrent treatment**	
Concurrent treatment improves BHCs.	63.68 [221]
People with non-nicotine SUDs should quit using other drugs/tobacco concurrently.	32.85 [114]
**Provider TFWP value**	
TFWPs provide clean and safe environments for staff and patients.	71.76 [249]
TFWPs help patients and staff quit smoking.	62.25 [216]
**Past-year training on concurrent treatment**	12.97 [45]

Note. BHCs = behavioral health conditions; SUDs = substance use disorders; TFWPs = tobacco-free workplace policies.

**Table 4:** Associations between providers’ attitudes on concurrent treatment of tobacco use and behavioral health disorders with their perceived value of tobacco-free workplace policies in Texas healthcare centers serving rural and/or medically underserved areas (*N* = 347).

	No	Yes					*p*-Value
	% [*n*]		Adjusted Estimate	OR	95% CI	ICC	
**Attitude 1** **. Concurrent treatment improves BHCs.**							
TFWPs provide clean and safe environments for staff and patients.	53.97 [68]	81.90 [181]	1.34	3.81	(2.31, 6.28)	0.0004	<0.001
TFWPs help patients and staff quit smoking.	45.24 [57]	71.95 [159]	1.11	3.04	(1.90, 4.87)	0.0187	<0.001
**Attitude 2. People with non-nicotine SUDs should quit using other drugs/tobacco concurrently.**							
TFWPs provide clean and safe environments for staff and patients.	69.96 [163]	75.44 [86]	0.22	1.25	(0.74, 2.11)	<0.0001	0.408
TFWPs help patients and staff quit smoking.	57.51 [134]	71.93 [82]	0.57	1.77	(1.08, 2.92)	0.0034	0.024

Note. TFWPs = tobacco-free workplace policies; SUDs = substance use disorders; BHCs = behavioral health conditions. Analyses controlled for TFWP existence at the healthcare center level and past-year training receipt. OR = odds ratio; CI = confidence interval; ICC = intraclass correlation coefficient. Generalized linear mixed models were used. TFWP existence at the healthcare center level was controlled, and a nested data structure was considered.

**Table 5:** Qualitative analysis: summary of the themes and findings on staff attitudes on concurrent treatment of tobacco use and behavioral health disorders in Texas healthcare centers serving rural and/or medically underserved areas.

Themes	Subthemes and Findings
Prioritization of “harm reduction” techniques	Participants expressed a need to connect with their patients in ways that emphasize harm reduction. These perceptions included: • Patients may not be willing or able to quit both non-nicotine substances and tobacco at the same time. • Concurrent treatment could negatively affect their BHC treatment. • Patients should initiate discussions about tobacco cessation and establish their own treatment goals.
Limited support for providing tobacco use screenings and tobacco cessation services	Many participants indicated that their HCs had limited education and staffing for implementing tobacco use screening and cessation services. Participants stated: • Providers are interested in acquiring tobacco use treatment education to increase capacity for providing these services. • HCs do not have the necessary resources to provide tobacco use counseling/services. • Some participants were unaware of or underutilized easily accessible resources like the Texas Tobacco Quitline.
Hesitation about tobacco-free workplace program integration	Participants expressed concern about integrating tobacco-free workplace programs into their HCs because: • TFWPs may stigmatize patient behaviors and infringe on their bodily autonomy. • Patients may use tobacco to cope with stress. • Patients might not maintain reduction/cessation after completing the substance use treatment program due to decreased access to NRT.

Note. BHC = behavioral health condition; HCs = healthcare centers; NRT = nicotine replacement therapy; TFWPs = tobacco-free workplace policies.

## Data Availability

The data from this study can be requested from the corresponding author. They are not publicly accessible due to funder restrictions and ongoing reporting of outcome papers from the dataset.

## Supplementary Materials

The following supporting information can be downloaded at: https://www.mdpi.com/article/10.3390/healthcare14203482/s1. File S1: Pre-implementation staff survey. Table S1: Correlations between providers’ attitudes toward concurrent treatment of tobacco use and behavioral health disorders and their perceived value of tobacco-free workplace policies in rural and/or medically underserved areas of Texas.

## Abbreviations

The following abbreviations are used in this manuscript:

BHC	Behavioral health condition
CI	Confidence interval
HCs	Healthcare centers
ICC	Intraclass correlation coefficient
LMHA	Local mental health authorities
MHCs	Medical healthcare centers
MUAs	Medically underserved areas
NRT	Nicotine replacement therapy
OR	Odds ratios
ORC	Organizational readiness for change
QIAB	Quality Improvement Assessment Board
RQA	Rapid qualitative analysis
SUDs	Substance use disorders
SUTCs	Substance use treatment centers
TFWP	Tobacco-free workplace policy
TRTTF	Taking Rural Texas Tobacco-Free
TTTF	Taking Texas Tobacco-Free
