# Supplementary material for: Evaluating Healthcare Center Staff Attitudes Toward Adoption of Tobacco-Free Policies and Concurrent Tobacco Use and Behavioral Health Treatment in Rural and Medically Underserved Areas of Texas
Source: Healthcare (Basel). 2026 Oct 16;14(20):3482. doi: 10.3390/healthcare14203482 (PMC13704411; doi:10.3390/healthcare14203482)
Supplement: Supplementary file 1 [file healthcare-14-03482-s001.zip › healthcare - File S1. Pre-Implementation Survey.pdf]

TAKING RURAL TEXAS TOBACCO FREE

@TTTF_ @TakingTexasTobaccoFree

www.takingtexastobaccofree.com

Screener

1a. What center do you work for?

Taking Rural Texas Tobacco Free

Pre-Implementation Survey

Purpose of Project

The goal of *Taking Rural Texas Tobacco Free* is to disseminate and implement a Tobacco-free Workplace Program to selected Substance Use Treatment Centers (SUTCs) and Health Centers (e.g., Federally Qualified Health Centers [FQHCs]) located within rural and medically underserved areas of Texas or serving patients

from rural and medically underserved areas of Texas. Your CEO has signed a Memorandum of Understanding indicating that your center will be participating in this project. Therefore, we are attempting to collect data that accurately reflects the current status of you center and its general (non-patient facing) employees and treatment providers before the program is implemented.

Purpose of this Survey

The *Taking Rural Texas Tobacco Free* project has various components and this survey is one of them. As part of this program, you are being asked to complete a brief survey that assesses various topic areas. For all employees, we are interested in information about your tobacco use status, quitting history, training experience, and thoughts about our program. For those identifying as treatment providers, we are interested in how you typically handle tobacco use in practice with your patients and what training you have received in this area. The survey is brief, and should take you about 2-20 minutes to complete. It will not be linked with your name in any way, so please be honest. The information gathered in this survey will help us to better understand the needs of your center so we can tailor our services for you. We will report final results to your center, but only in aggregate/grouped form (i.e., responses for ALL employees combined). We will also report this aggregate data to our funder, the Cancer Prevention & Research Institute of Texas, and may publish results in professional forums. Survey data will be maintained for at least three years following the completion of the project aims.

By completing the survey, you acknowledge that you: (a) have read the goal of the project, (b) have decided to complete the part of the project described here, and (c) understand that you will be contacted again in a few months to complete a similar survey (after *Taking Rural Texas Tobacco Free* has been implemented at your center); however, you will be free to refuse any further participation if you wish.

Your anonymous raw survey data will be shared with the project team at The University of Texas at MD Anderson Cancer Center and the University of Houston, who will put all the information together in report form. By answering the questions, you are providing authorization for the project team to use and share your anonymous information as part of this project. Please note that your decision to participate (or not) will not be shared with your employer. Should you choose to participate, your individual responses to this survey will not be shared with your employer.

Please use the link provided at the end of this survey to enter your contact information and receive the $10 Amazon e-gift card. Your contact information will be kept separate from your responses to this survey to maintain your anonymity. **Please note we are only seeking 1 response per employee and only the first response from each employee will be considered for compensation.**

Thank you for your assistance. If you have questions about this survey, please contact Lorraine Reitzel (LReitzel@mdanderson.org) of The University of Texas at MD Anderson Cancer Center. You can read more about some of our prior work at www.TakingTexasTobaccoFree.com.

Any questions regarding your rights as a research subject may be addressed to the University of Houston Institutional Review Board (713-743-9204).

Pre-Implementation Survey

Purpose of Project

The goal of *Taking Rural Texas Tobacco Free* is to disseminate and implement a Tobacco-free Workplace Program to selected Substance Use Treatment Centers (SUTCs) and Health Centers (e.g., Federally Qualified Health Centers [FQHCs]) located within rural and medically underserved areas of Texas or serving patients from rural and medically underserved areas of Texas. Your CEO has signed a Memorandum of Understanding indicating that your center will be participating in this project. Therefore, we are attempting to collect data that accurately reflects the current status of you center and its general (non-patient facing) employees and treatment providers before the program is implemented.

Purpose of this Survey

The *Taking Rural Texas Tobacco Free* project has various components and this survey is one of them. As part of this program, you are being asked to complete a brief survey that assesses various topic areas. For all employees, we are interested in information about your tobacco use status, quitting history, training experience, and thoughts about our program. For those identifying as treatment providers, we are interested in how you typically handle tobacco use in practice with your patients and what training you have received in this area. The survey is brief, and should take you about 2-20 minutes to complete. It will not be linked with your name in any way, so please be honest. The information gathered in this survey will help us to better understand the needs of your center so we can tailor our services for you. We will report final results to your center, but only in aggregate/grouped form (i.e., responses for ALL employees combined). We will also report this aggregate data to our funder, the Cancer Prevention & Research Institute of Texas, and may publish results in

professional forums. Survey data will be maintained for at least three years following the completion of the project aims.

By completing the survey, you acknowledge that you: (a) have read the goal of the project, (b) have decided to complete the part of the project described here, and (c) understand that you will be contacted again in a few months to complete a similar survey (after *Taking Rural Texas Tobacco Free* has been implemented at your center); however, you will be free to refuse any further participation if you wish.

Your anonymous raw survey data will be shared with the project team at The University of Texas at MD Anderson Cancer Center and the University of Houston, who will put all the information together in report form. By answering the questions, you are providing authorization for the project team to use and share your anonymous information as part of this project. Please note that your decision to participate (or not) will not be shared with your employer. Should you choose to participate, your individual responses to this survey will not be shared with your employer.

Thank you for your assistance. If you have questions about this survey, please contact Lorraine Reitzel (LReitzel@mdanderson.org) of The University of Texas at MD Anderson Cancer Center. You can read more about some of our prior work at www.TakingTexasTobaccoFree.com.

Any questions regarding your rights as a research subject may be addressed to the University of Houston Institutional Review Board (713-743-9204).

Click to write the question text

I'm not a robot

reCAPTCHA
Privacy - Terms

Click to write the question text

Click here if you consent to participate and to complete this survey

Center

Please choose the location(s) where you work. If your location is not listed, please use the last option.

1. Blanco County Mental Health – 206 S Hwy 281, Johnson City, TX 78636
2. Bulverde Mental Health – 1781 E Ammann Rd, Bulverde, TX 78163
3. Canyon Lake Mental Health – 230 Shepherd Hill Dr, Canyon Lake, TX, TX 78133
4. Comal County Mental Health – 358 Landa Street, Suite 300, New Braunfels, TX 78130

5. Gillespie County Mental Health – 140 Industrial Loop, Suite 200, Fredericksburg, TX 78624

6. Hays County Mental Health (Scheib Center) – 1200 N Bishop St, San Marcos, TX, 78666

7. Kendall County Mental Health – 221 Fawn Valley Dr, Suite 500, Boerne, TX 78006

8. Kerr County Mental Health – 955 Water Street, Kerrville, TX 78028

9. Kimble County Mental Health – 110 S 10th St, Junction, TX 76849

10. Kyle Mental Health – 1300 Dacy Lane, Suites 120-130, Kyle, TX 78640

11. Llano County Mental Health – 100 Legend Hills, Suite B, Llano, TX 78643

12. Medina County Mental Health – 728 18th St, Hondo, TX 78861

13. Uvalde County Mental Health – 328 Crystal City Hwy, Uvalde, TX 78801

14. Val Verde County Mental Health – 1927 N Bedell, Del Rio, TX 78840

15. **My location not listed above.**

Are you involved in the provision of dental services (e.g., dentists and dental hygienists)?

Yes

No

Which location are you employed at?

Mission Clinic

San Juan Clinic

Other (please specify):

Are you involved in the provision of dental services (e.g., dentists and dental hygienists)?

Yes

No

Which location are you employed at?

PHNTX Dental Care - East Dallas

PHNTX Dental Care - Farmers Branch

PHNTX Dental Care - Vickery Meadow

Default Question Block

1b. Are you a full-time employee?

Yes

No

N/A, I'm a volunteer provider from a partner hospital

2. How often do you smoke cigarettes (choose the best answer)?

Every day

Some days

Weekends

Only when I drink alcohol

I do not smoke or have quit completely

3. Have you ever routinely smoked more cigarettes per day than you are smoking now?

Yes

No

4. On how many of the past 30 days did you smoke cigarettes? (Days = 0-30)

5. On the days that you smoke how many cigarettes do you smoke each day? (Number of cigarettes)

6. Have you used any of the following other/alternative tobacco products more than 1 time within the last year? (Click ALL items that apply)

a. Snus, such as Camel or Marlboro Snus

b. Roll your own cigarettes

c. Tobacco from a hookah or a waterpipe

d. Dissolvable tobacco products like Ariva/Stonewall/Camel/Camel Orbs/Camel sticks

e. Electronic cigarettes or E-cigarettes, (including battery operated vape pens, e-pipes, e-cigars, personal vaporizers, or ehookahs), such as Fin, NJOY, Blu, e-Go, and Vuse

f. Cigars

g. Little cigars/cigarillos/bidi's

h. Chewing tobacco, dip, or snuff

i. Other tobacco products (besides conventional cigarettes, please specify)

j. I have not used any other/alternative tobacco products in the past year

7. Would you be interested in quitting cigarettes and/or other nicotine/tobacco products if cessation services (e.g., counseling, support groups, nicotine replacement therapy) were offered to you free of charge at your workplace?

Yes

No

Maybe

8. Have you ever used any resources offered on the *Taking Texas Tobacco Free* website (www.takingtexastobaccofree.com)?

Yes

No

I'm not sure

9. Please indicate your level of agreement or disagreement with the following statements.

People with mental health and/or non-nicotine substance use disorders...

	Strongly disagree	Somewhat disagree	Neither agree nor disagree	Somewhat agree	Strongly agree
a. ...are approximately twice as likely as the general population to smoke cigarettes	○	○	○	○	○
b. ...are more likely to die from smoking-related illnesses than from their mental and/or non-nicotine substance use disorder	○	○	○	○	○
c. ...who smoke want to quit smoking and are able to quit smoking	○	○	○	○	○

	Strongly disagree	Somewhat disagree	Neither agree nor disagree	Somewhat agree	Strongly agree
d. ...who quit smoking may experience concurrent improvements in some mental health symptoms and/or reductions in non-nicotine substance use	○	○	○	○	○

10. In the last 12 months, have you received any education at (or sponsored by) your center regarding the hazards of smoking?

Yes

No

11. In the last 12 months, have you received any training at (or sponsored by) your center regarding the hazards of smoking and benefits of quitting that are specific to individuals with substance use disorders?

Yes

No

12. How is tobacco use handled on the premises of your workplace?

a. Tobacco use is freely allowed inside and outside buildings/residences

b. Tobacco use is freely allowed outside buildings/residences

c. Tobacco use is restricted to a designated area on the property

d. Tobacco use is disallowed indoors and on our property

e. Other

13. Does your center have a tobacco-free workplace policy?

Yes

No

I do not know

14. Are exceptions to your tobacco use policy allowed for any of the following groups? Please endorse groups for whom exceptions are made

(check all that apply):

a. Contractors

b. Visitors

c. Inpatient/residential patients

d. Employees

e. There are no groups/individuals for whom exceptions are made

f. I have no idea what exceptions are in the policy

g. Other

15. Please indicate your level of agreement or disagreement with the following statements.

	Strongly disagree	Somewhat disagree	Neither agree nor disagree	Somewhat agree	Strongly agree
a. My center has clear signage regarding our tobacco use policy	○	○	○	○	○
b. My center ensures that the tobacco use policy is followed	○	○	○	○	○

	Strongly disagree	Somewhat disagree	Neither agree nor disagree	Somewhat agree	Strongly agree
c. Our tobacco use policy is consistently enforced	○	○	○	○	○
d. Our tobacco use policy is enforced in a fair manner	○	○	○	○	○
e. Our patients are aware of our tobacco use policy	○	○	○	○	○
f. Our contractors are aware of our tobacco use policy	○	○	○	○	○
g. Our visitors are aware of our tobacco use policy	○	○	○	○	○

16. What are your concerns about sustaining or maintaining a tobacco-free workplace policy? (Click ALL items that apply).

a. I am unsure that my center completely supports the policy

b. I am concerned about complaints from consumers

c. I am concerned that it upsets my colleagues who smoke

d. My center lacks the resources to adequately enforce the policy

e. My center may experience a reduced demand for our clinical services

f. Other (please specify)

g. I do not have any significant concerns about maintaining a tobacco-free workplace policy.

17. Are you a direct service provider who provides treatment to patients aged 16 or older at your workplace? We define a direct service provider as someone who typically has one or more of the following credentials (and other similar credentials): NP, LVN, RN, APN, CNA, MA, QMHP, MD, LCDC, LSW, etc. Direct service providers might also have titles like recovery coach, patient navigator, or peer support specialist.

Yes

No

18. About how many patients have you seen over the last month aged 16 years or older?

19. About how many of the patients that you have seen over the last month aged 16 years or older were **new** patients for you?

20. About how many of the patients that you have seen over the last month aged 16 years or older were conventional cigarette smokers?

21. About how many of the patients that you have seen over the last month aged 16 years or older used other (non-cigarette) tobacco products (including but not limited to e-cigarettes or vaping products)?

22. Please indicate your level of agreement or disagreement with the following statements.

	Strongly disagree	Somewhat disagree	Neither agree nor disagree	Somewhat agree	Strongly agree
a. I have the required skills to help my patients quit smoking	○	○	○	○	○
b. My patients are concerned about smoking	○	○	○	○	○
c. My patients follow my advice about behavior change	○	○	○	○	○
d. My patients who smoke want to quit smoking	○	○	○	○	○

	Strongly disagree	Somewhat disagree	Neither agree nor disagree	Somewhat agree	Strongly agree
e. I know where to refer patients for help with smoking cessation	○	○	○	○	○

23. Please indicate your level of agreement or disagreement with the following statements.

	Strongly disagree	Somewhat disagree	Neither agree nor disagree	Somewhat agree	Strongly agree
a. I have the required skills to help my patients quit other (non-cigarette) tobacco use (e.g., vaping products, smokeless tobacco, etc.)	○	○	○	○	○
b. My patients are concerned about other (non-cigarette) tobacco use	○	○	○	○	○

	Strongly disagree	Somewhat disagree	Neither agree nor disagree	Somewhat agree	Strongly agree
c. My patients who use other (non-cigarette) tobacco products want to quit	○	○	○	○	○
d. I know where to refer patients for help with quitting other (non-cigarette) tobacco use	○	○	○	○	○

24. You said you saw ${q://QID55/ChoiceTextEntryValue} **new** patients aged 16 years or older in the last month. How many of these patients were given a comprehensive Tobacco Use Assessment (includes information like patient’s smoking status, smoking history, cigarettes, or packs smoked per day, number of years smoked, number of years since quitting smoking, etc.) ?

[ ]

25. You said you saw ${q://QID54/ChoiceTextEntryValue} patients aged 16 years or older in the last month. How many of these patients did you:

a. Ask whether they smoked cigarettes?

b. Ask whether they used other (non-cigarette) tobacco products (including but not limited to e-cigarettes and vapes)?

26. You said you saw ${q://QID56/ChoiceTextEntryValue} patients aged 16 years or older who were conventional cigarette smokers in the last month. How many of these patients did you:

a. Advise to quit smoking?

b. Assess interest in making a smoking quit attempt?

c. Assist to make a quit attempt (e.g., referral to the Texas Tobacco Quitline, on-site or offsite referrals, provided a direct intervention like counseling, medication, NRT)?

d. Arrange a follow-up contact to discuss progress with quitting?

27. You said you saw ${q://QID57/ChoiceTextEntryValue} patients aged 16 years or older who were users of other (non-cigarette) tobacco products in the last month (including but not limited to e-cigarettes and vapes). How many of these patients did you:

a. Advise to quit?

b. Assess interest in making a quit attempt?

c. Assist to make a quit attempt (e.g., referral to the Texas Tobacco Quitline, on-site or offsite referrals, provided a direct intervention like counseling, medication, NRT)?

d. Arrange a follow-up contact to discuss progress with quitting?

28. You said you saw ${q://QID56/ChoiceTextEntryValue} patients aged 16 years or older who were smokers in the last month. Of those smokers who indicated they were not interested in quitting smoking, with how many of these patients did you:

a. Use brief Motivational Interviewing

b. Use the "5 R's": Relevance, Risks, Rewards, Roadblocks, and Repetition. click here for more information on the 5 Rs

c. Advise to consider quitting in the future

d. Provide with tobacco-related health promotion materials

e. Tell you would revisit this at the next contact

f. Provide the Texas Tobacco Quitline contact information

g. Provide an intervention not listed above

h. Provide no intervention

29. You said you saw ${q://QID57/ChoiceTextEntryValue} patients aged 16 years or older who were users of other (non-cigarette) tobacco products in the last month (including but not limited to e-cigarettes and vapes). Of those users who indicated they were not interested in quitting non-cigarette tobacco products, with how many of these patients did you:

a. Use brief Motivational Interviewing

b. Use the "5 R's": Relevance, Risks, Rewards, Roadblocks, and Repetition. click here for more information on the 5 Rs

c. Advise to consider quitting in the future

d. Provide with tobacco-related health promotion materials

e. Tell you would revisit this at the next contact

f. Provide the Texas Tobacco Quitline contact information

g. Provide an intervention not listed above

h. Provide no intervention

30. In the last month, how frequently did you encourage your patients aged 16 years or older who smoke cigarettes to:

	Never	Sometimes	About half the time	Most of the time	Always
a. Stop smoking completely	○	○	○	○	○
b. Use nicotine replacement	○	○	○	○	○

	Never	Sometimes	About half the time	Most of the time	Always
c. Reduce smoking to 5 or fewer cigarettes per day, if patient stated they could not quit	○	○	○	○	○
d. Not smoke in their home and car	○	○	○	○	○
e. Not smoke in the presence of infants or children	○	○	○	○	○
f. Consider switching to e-cigarettes or smokeless tobacco products	○	○	○	○	○

31. In the last month, how frequently did you encourage your patients aged 16 years or older who use tobacco products other than cigarettes (e.g., snuff, dip, e-cigarettes, snus) to:

	Never	Sometimes	About half the time	Most of the time	Always
a. Stop other (non-cigarette) tobacco product use completely	○	○	○	○	○
b. Use nicotine replacement	○	○	○	○	○
c. Reduce (non-cigarette) tobacco use, if patient stated they could not quit	○	○	○	○	○
d. Not use (non-cigarette) tobacco products in their home and car	○	○	○	○	○
e. Not use (non-cigarette) tobacco products in the presence of infants or children	○	○	○	○	○

32. Please clarify how knowledgeable you are about how to obtain reimbursements for smoking/tobacco cessation services from patients' public and/or private insurers?

Not at all knowledgeable

Slightly knowledgeable

Moderately knowledgeable

Very knowledgeable

Extremely knowledgeable

33. Do you routinely seek insurance reimbursement for the smoking/tobacco cessation services you provide to your patients?

Yes

No

I do not know

34. Does your center encourage you to seek reimbursement for smoking cessation counseling from patients’ private or public insurance coverage?

Yes

No

I do not know

35. In your opinion, how prepared are the direct service providers in your workplace to help their patients aged 16 years or older quit tobacco use?

- Not at all prepared to do so
- Somewhat unprepared to do so
- Neither prepared nor unprepared to do so
- Somewhat prepared to do so
- Completely prepared to do so

36. Please tell us what might be needed, in your opinion, to better prepare direct service providers to help their patients quit tobacco use.

37. Please indicate your level of agreement or disagreement with the following statements.

	Strongly disagree	Somewhat disagree	Neither agree nor disagree	Somewhat agree	Strongly agree
a. Smoking cessation counseling is an important part of my job	○	○	○	○	○
b. (Non-cigarette) tobacco use cessation counseling is an important part of my job	○	○	○	○	○
c. I am able to tailor cessation counseling to my patient’s needs	○	○	○	○	○
d. Smoking cessation/tobacco use counseling is not applicable to the patients I serve	○	○	○	○	○
e. If a patient has been in recovery from alcoholism for <6 months, quitting smoking would threaten their sobriety	○	○	○	○	○
f. Tobacco cessation counseling is an important part of my center's mission	○	○	○	○	○

	Strongly disagree	Somewhat disagree	Neither agree nor disagree	Somewhat agree	Strongly agree
g. Direct service providers should advise patients to quit even if that is not the reason for the visit	○	○	○	○	○
h. Counseling by a direct service provider helps motivate smokers and other tobacco product users to quit	○	○	○	○	○
i. Direct service providers should make appointments to specifically help patients quit smoking or using other tobacco products	○	○	○	○	○
j. Smoking or other tobacco product use is a personal decision that does not concern the direct service provider	○	○	○	○	○
k. Tobacco free workplace policies are important because they provide a clean and safe environment for our staff to work in and patients to receive care in	○	○	○	○	○

	Strongly disagree	Somewhat disagree	Neither agree nor disagree	Somewhat agree	Strongly agree
l. Tobacco free workplace policies may help our patients and employees quit smoking	○	○	○	○	○
m. Tobacco use cessation is not a feasible goal for our patients	○	○	○	○	○
n. Tobacco use contributes significantly to our property maintenance costs	○	○	○	○	○
o. I would be willing to be trained to offer tobacco cessation counseling to our patients	○	○	○	○	○
p. Sometimes it is useful for staff to smoke with patients as a way of building trust and rapport	○	○	○	○	○
q. I feel confident in my ability to offer smoking cessation counseling to patients	○	○	○	○	○

38. Please select any or all of the following statements that may be true based on practices in your center over the last couple of years (select all that apply).

a. I have seen staff smoke with patients on smoking breaks

b. I have seen staff offer patients cigarettes

c. I sometimes have smoked with patients (e.g., to build a therapeutic relationship with them, etc.)

39. In your opinion, what is the best point to encourage patients to stop smoking or using other tobacco products?

As soon as they begin treatment

After 1 year of treatment

It depends on the patient

Never

40. In your opinion, for patients who use illicit drugs and tobacco products (and are open to any of the options below), which should come

first?

- Quit using drugs
- Quit using tobacco
- Quit using drugs and tobacco at the same time

41. Does your center have at least 1 professional on site who can prescribe tobacco cessation medications (e.g., Chantix, Zyban)?

- Yes
- No
- I do not know

42. Does your center have an on-site pharmacy?

- Yes
- No
- I do not know

43. Does your on-site pharmacy sell nicotine replacement therapies?

Yes

No

I do not know

44. Has your center ever offered nicotine replacement therapies free of charge to patients?

Yes

No

I do not now

45. Does your center (i.e., the location/clinic where you work) employ at least 1 person who is considered an expert on treating tobacco (e.g., someone who completed Certified Tobacco Treatment Specialist training)?

Yes

No

I do not know

46. To your knowledge, does your center provide telehealth services to patients?

Yes

No

I do not know

47. Does your center mandate that every patient aged 16 years or older is screened for tobacco use at intake and that this is documented in the patient record?

Yes

No

I do not know

48. After intake and upon admission into your treatment program, what is the next step in addressing patients' tobacco use?

a. Tobacco use is noted in the intake assessment and is the responsibility of the direct service provider to address it with the patient

b. It is up to the patient to decide whether they want help quitting. *Patients are asked if they want help*, and this information is passed along to the direct service provider

c. It is up to the patient to decide whether they want help quitting; *if they want help, patients need to know to ask for help* - this information is then passed along to the direct service provider

d. It is the direct service provider's responsibility to identify whether the patient is a tobacco user and address it at the direct service provider's discretion

e. We have no formal process of assessing for tobacco use at intake

f. Other

49. In the last 12 months, have you attended any trainings that covered the following topics :

	Yes	No	I do not know
a. How to screen/assess patients for tobacco use?	○	○	○
b. How to treat patients for tobacco use?	○	○	○
c. How to motivate a patient to quit tobacco use?	○	○	○

	Yes	No	I do not know
d. How to treat tobacco use concurrently with non-nicotine substance use/abuse?	○	○	○
e. How to use counseling and other behavioral therapies (e.g., Motivational Interviewing) to treat tobacco use?	○	○	○
f. How quitting tobacco use may impact the effectiveness of other medications?	○	○	○

50. Does your center offer continuing education about tobacco use and cessation (e.g., in service trainings, annual trainings) to direct service providers and/or all employees?

Yes, but to direct service providers only

Yes, to all employees

No

I do not know

51. Listed below are various things that can limit your center's capacity to offer smoking or tobacco use cessation counseling. If any of these are applicable to your center, please rate how important you think it is in limiting your center's capacity to offer tobacco cessation services to patients.

	Not at all important	Slightly important	Moderately important	Very important	Extremely important	N/A
a. Patients are not interested	○	○	○	○	○	○
b. Patients do not comply with treatment	○	○	○	○	○	○
c. Center leadership is not interested/invested in this	○	○	○	○	○	○
d. Employees are not interested/invested in doing this	○	○	○	○	○	○
e. Employees do not know how to do this or lack confidence in their abilities to do this	○	○	○	○	○	○

	Not at all important	Slightly important	Moderately important	Very important	Extremely important	N/A
f. Lack of impact on patients	○	○	○	○	○	○
g. Lack of time for direct service providers to provide tobacco cessation services	○	○	○	○	○	○
h. Lack of reimbursement/it costs too much to do this	○	○	○	○	○	○
i. Lack of community resources to refer patients	○	○	○	○	○	○
j. Lack of patient education material	○	○	○	○	○	○
k. Lack of training	○	○	○	○	○	○
l. Complexity of smoking or other tobacco use cessation guidelines	○	○	○	○	○	○
m. Importance of competing problems/diagnoses/comorbidities	○	○	○	○	○	○

	Not at all important	Slightly important	Moderately important	Very important	Extremely important	N/A
n. Other	○	○	○	○	○	○

52. Does your center use an electronic health record with a hard stop that requires the entry information about patient smoking status?

Yes

No

N/A, we do not have an electronic health record but we require patient smoking status entry in paper records

N/A, we do not have an electronic health record and do not require patient smoking status entry in paper records

I do not know

53. In your opinion, what support or services (e.g., education/information, specialized training, materials, technical assistance for policy implementation, nicotine replacement) does your

center need in order to better or more comprehensively address tobacco use among your patients?

54. Do you receive any funding from the state or external funders that requires your center to certify that it meets or exceeds tobacco-free workplace minimums?

Yes

No

I do not know

55. Does your center provide a template or a guide for conducting a Tobacco Use Assessment that includes information like patient's smoking status, smoking history, cigarettes, or packs smoked per day, number of years smoked, number of years since quitting smoking, etc.?

Yes

No

I do not know

56. Do your center's healthcare providers assess for eligibility and/or refer current or former smokers who meet eligibility criteria to low-dose computed tomography (LDCT) for lung cancer screening?

Yes, we assess for eligibility but do NOT refer to LDCT

Yes, we assess for eligibility AND we refer to LDCT

No, we do not assess eligibility or refer to LDCT

I do not know

57. Please indicate your level of agreement or disagreement with the following statements:

	Strongly disagree	Somewhat disagree	Neither agree nor disagree	Somewhat agree	Strongly agree	N/A
a. My center suggests that we refer smokers and other tobacco users to the Texas Tobacco Quitline	○	○	○	○	○	○
b. The Texas Tobacco Quitline is helpful to patients who want to quit	○	○	○	○	○	○
c. I have had a good experience with the Texas Tobacco Quitline as a provider	○	○	○	○	○	○
d. My patients have had a good experience with the Texas Tobacco Quitline	○	○	○	○	○	○
e. I am unfamiliar with The Texas Tobacco Quitline	○	○	○	○	○	○

This is the final page of the survey. You can use the forward arrow to submit your response. Otherwise, you can use the back arrow to review

any of your responses.

Powered by Qualtrics
