# Supplementary material for: Evaluating Healthcare Center Staff Attitudes Toward Adoption of Tobacco-Free Policies and Concurrent Tobacco Use and Behavioral Health Treatment in Rural and Medically Underserved Areas of Texas
Source: Healthcare (Basel). 2026 Oct 16;14(20):3482. doi: 10.3390/healthcare14203482 (PMC13704411; doi:10.3390/healthcare14203482)
Supplement: Supplementary file 1 [file healthcare-14-03482-s001.zip › healthcare - Table S1.pdf]

Table S1. Correlations Between Providers' Attitudes Toward Concurrent Treatment of Tobacco Use and Behavioral Health Disorders and Their Perceived Value of Tobacco-Free Workplace Policies in Rural and/or Medically Underserved Areas of Texas.

Correlation coefficient (*p*-value)	1	2	3	4
1) Attitude 1. Concurrent treatment improves BHCs.		0.03 (0.52)	0.20 (<0.001)	0.16 (<0.01)
2) Attutide 2. People with non-nicotine SUDs should quit using other drugs/tobacco concurrently.			0.08 (0.16)	0.15 (<0.01)
3) TFWPs provide clean and safe environments for staff and patients.				0.70 (<0.001)
4) TFWPs help patients and staff quit smoking.				

Note. Values are correlation coefficients. BHCs = behavioral health conditions; SUDs = substance use disorders; TFWPs = tobacco-free workplace policies.
